# Supplementary material for: Single-sided magnetic resonance-based sensor for point-of-care evaluation of muscle
Source: Nat Commun. 2024 Jan 10;15:440. doi: 10.1038/s41467-023-44561-9 (PMC10782019; doi:10.1038/s41467-023-44561-9)
Supplement: Supplementary file 1 — Supplementary Information [file 41467_2023_44561_MOESM1_ESM.pdf]

Supplementary Materials for

Single-sided magnetic resonance-based sensor for point-of-care evaluation of muscle

Sydney E. Sherman, Alexa S. Zammit, Won-Seok Heo, Matthew S. Rosen, Michael J. Cima*

*Corresponding author. *E-mail address*: mjcima@mit.edu (M. J. Cima)

The PDF file includes:

Supplementary Figure 1: COMSOL Representation of magnetic field profile
Supplementary Figure 2: Permanent magnet array fabrication process
Supplementary Figure 3: Mapped permanent magnetic field
Supplementary Figure 4: T2 time comparison between single-sided sensors
Supplementary Figure 5: Effect of SNR on fit results
Supplementary Figure 6: Body and sensitive region position.

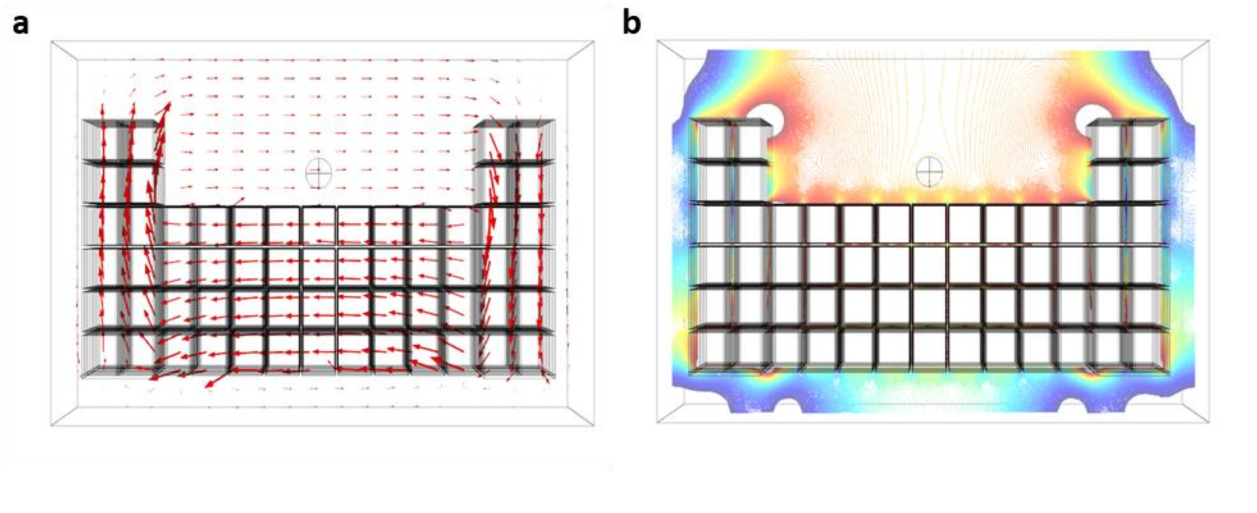

Supplementary Figure 1: COMSOL representations of magnetic field profile. a) arrows indicate direction of magnetic flux b) contour lines indicate strength of magnetic field

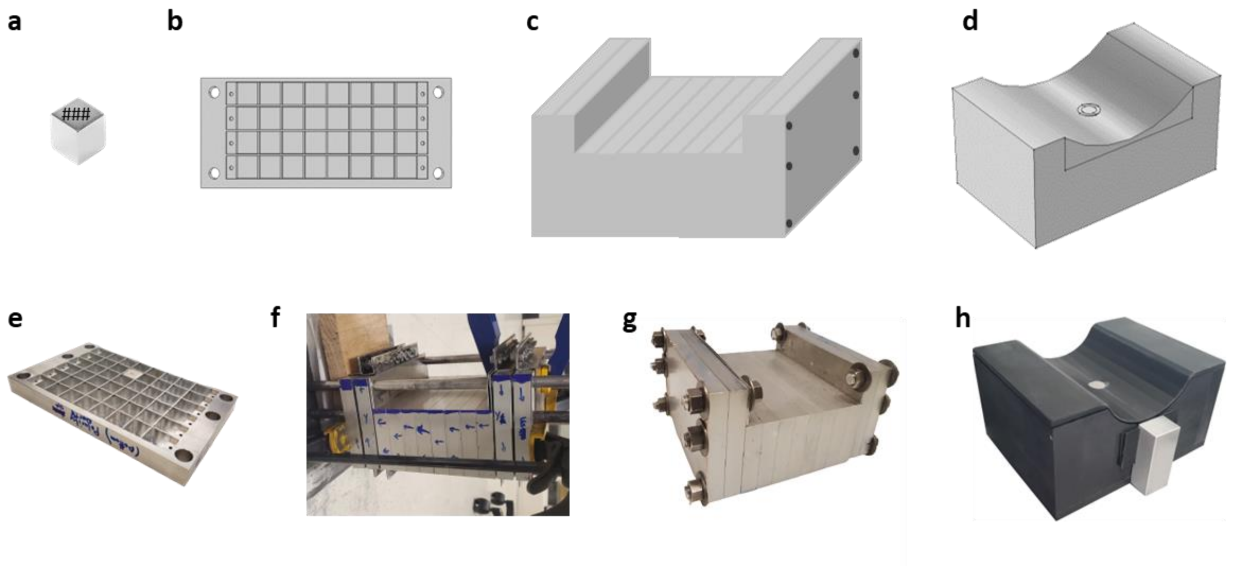

Supplementary Figure 2: Permanent magnet array fabrication process. a) 12.7mm cube magnet with serial number b) CAD rendering of aluminum frame for securing magnets c) Form of 12 frames secured together d) From of final casing surrounding the magnet array e) Picture of machined frame holding one cube magnet f) picture of the assembled magnet array with aluminum rods to secure the frames until bolts are placed g) Picture of secured magnet array h) Picture of final magnet array in Delrin casing with coil (center circle) and matching network (gray box secured to front of Delrin casing)

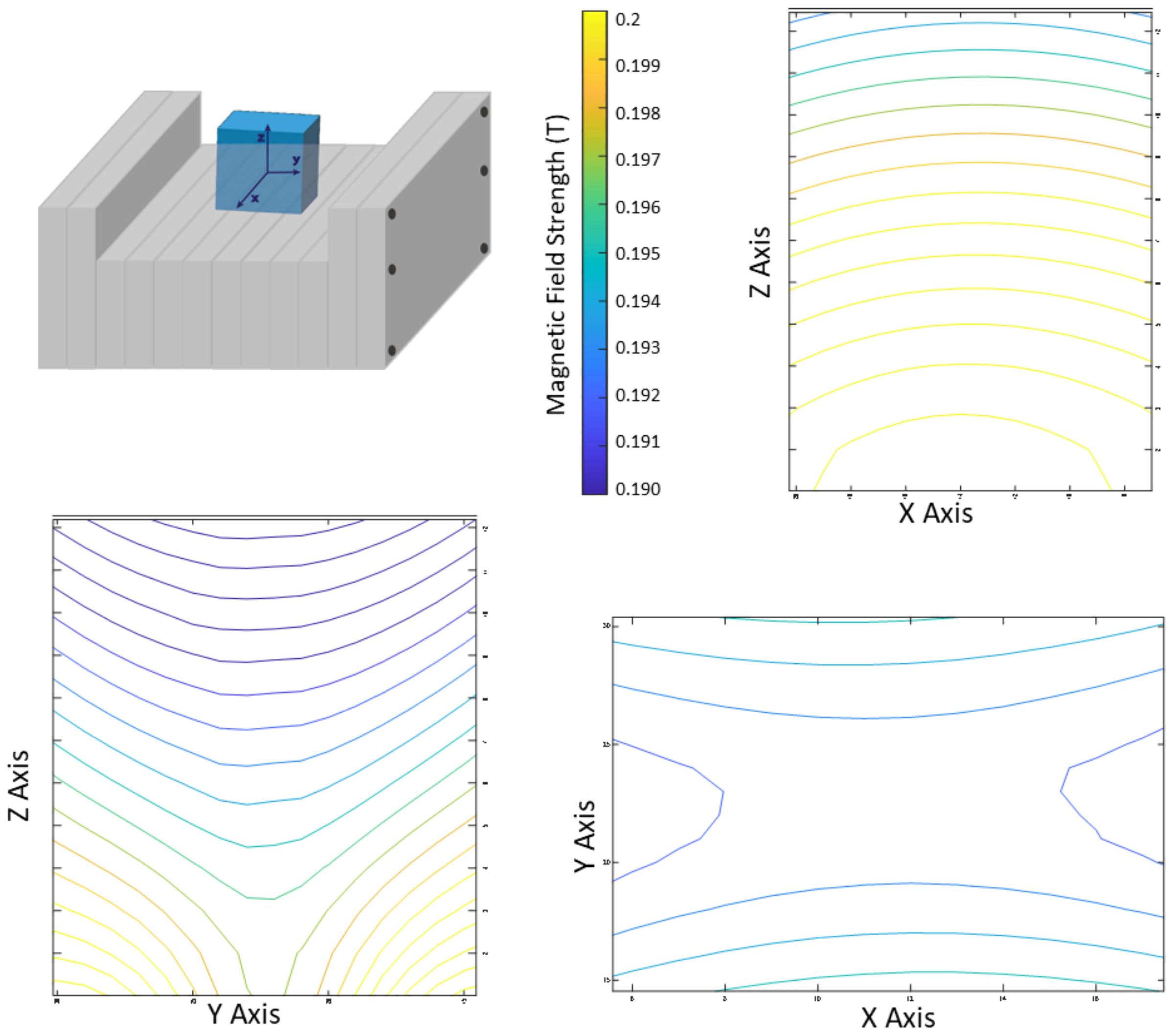

Supplementary Figure 3: Mapped permanent magnetic field. Representative cross sections of mapped permanent field along each axis.

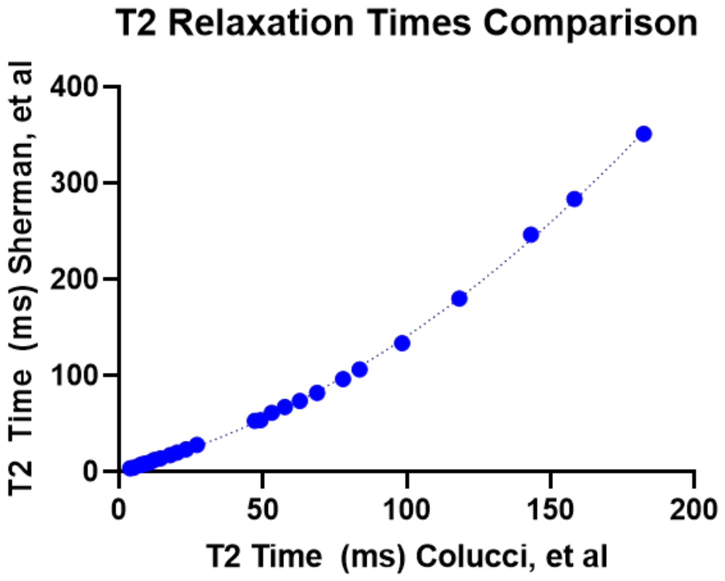

Supplementary figure 4: T2 time comparison between single-sided sensors. Relation between T2 times of 25 individual copper sulfate dilutions from data acquired from the magnet described in this manuscript and the magnet described in Colucci et al. Each copper sulfate phantom signal was acquired for 10 minutes to meet a minimum SNR threshold.

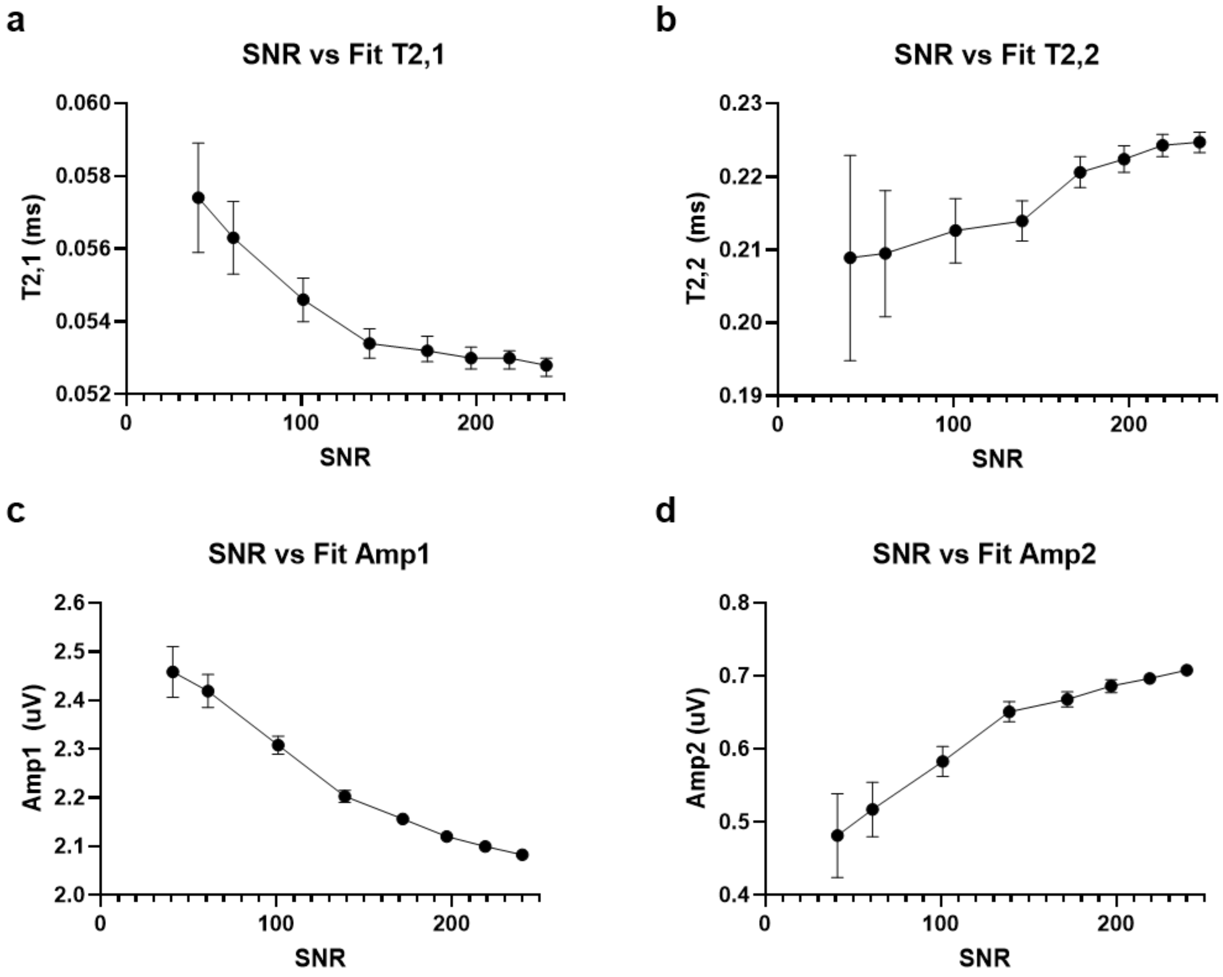

Supplementary figure 5. Effect of SNR on fit results. Bi-exponential fit results for muscle phantom a) T2,1 b) T2,2 c) Amp1, and d) Amp2 with increasing SNR. Error bars indicate 95% confidence interval of fit.

a b

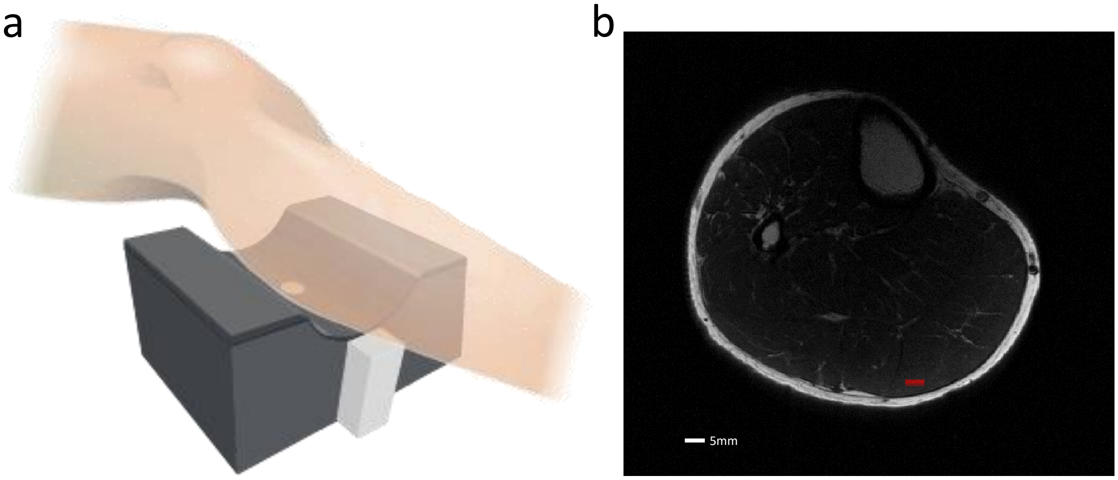

Supplementary figure 6. Body and sensitive region position. a) Calf positioning on sensor b) MRI of lower leg. Red mark indicates the position and size of the excited slice in muscle tissue.

Supplementary References

Figure S4:
Colucci, L. *et al.* Fluid assessment in dialysis patients by point-of-care magnetic relaxometry. *Sci Transl Med* **11**, (2019).
