# Supplementary material for: Single-sided magnetic resonance-based sensor for point-of-care evaluation of muscle
Source: Nat Commun. 2024 Jan 10;15:440. doi: 10.1038/s41467-023-44561-9 (PMC10782019; doi:10.1038/s41467-023-44561-9)
Supplement: Supplementary file 3 — Reporting Summary [file 41467_2023_44561_MOESM3_ESM.pdf]

nature portfolio

Corresponding author(s): Michael Cima

Last updated by author(s): Mar 11, 2023

Reporting Summary

Nature Portfolio wishes to improve the reproducibility of the work that we publish. This form provides structure for consistency and transparency in reporting. For further information on Nature Portfolio policies, see our Editorial Policies and the Editorial Policy Checklist.

Statistics

For all statistical analyses, confirm that the following items are present in the figure legend, table legend, main text, or Methods section.

n/a	Confirmed	
☐	☒	The exact sample size (*n*) for each experimental group/condition, given as a discrete number and unit of measurement
☐	☒	A statement on whether measurements were taken from distinct samples or whether the same sample was measured repeatedly
☐	☒	The statistical test(s) used AND whether they are one- or two-sided<br>*Only common tests should be described solely by name; describe more complex techniques in the Methods section.*
☒	☐	A description of all covariates tested
☒	☐	A description of any assumptions or corrections, such as tests of normality and adjustment for multiple comparisons
☐	☒	A full description of the statistical parameters including central tendency (e.g. means) or other basic estimates (e.g. regression coefficient) AND variation (e.g. standard deviation) or associated estimates of uncertainty (e.g. confidence intervals)
☐	☒	For null hypothesis testing, the test statistic (e.g. *F*, *t*, *r*) with confidence intervals, effect sizes, degrees of freedom and *P* value noted<br>*Give P values as exact values whenever suitable.*
☒	☐	For Bayesian analysis, information on the choice of priors and Markov chain Monte Carlo settings
☒	☐	For hierarchical and complex designs, identification of the appropriate level for tests and full reporting of outcomes
☒	☐	Estimates of effect sizes (e.g. Cohen's *d*, Pearson's *r*), indicating how they were calculated

Our web collection on statistics for biologists contains articles on many of the points above.

Software and code

Policy information about availability of computer code

Data collection	Prospa (Magritek, New Zealand, V3.100) software was used for data collection
Data analysis	MATLAB 2022b and Microsoft Excel were used for data analysis, no custom algorithms were used to data processing or analysis. Analysis code is accessible at: 10.6084/m9.figshare.24716349.

For manuscripts utilizing custom algorithms or software that are central to the research but not yet described in published literature, software must be made available to editors and reviewers. We strongly encourage code deposition in a community repository (e.g. GitHub). See the Nature Portfolio guidelines for submitting code & software for further information.

Data

Policy information about availability of data

All manuscripts must include a data availability statement. This statement should provide the following information, where applicable:

- Accession codes, unique identifiers, or web links for publicly available datasets
- A description of any restrictions on data availability
- For clinical datasets or third party data, please ensure that the statement adheres to our policy

Data has been placed in a public figshare repository, accessible at: 10.6084/m9.figshare.24716313. Source data is provided for all plots. Any additional requests for information can be directed to, and will be fulfilled by, the corresponding authors

Research involving human participants, their data, or biological material

Policy information about studies with human participants or human data. See also policy information about sex, gender (identity/presentation), and sexual orientation and race, ethnicity and racism.

Reporting on sex and gender	Sex and gender are not relevant to the outcomes of this study and were not considered in study design. Sex and gender are not expected to be relevant to study findings and this information was not collected. No analyses were completed based on sex or gender and subjects were recruited without regard to sex or gender.
Reporting on race, ethnicity, or other socially relevant groupings	There is no reporting of race, ethnicity, or other socially relevant grouping in the study. Subjects were recruited irrespective of race, ethnicity, or other socially relevant grouping and this information was not collected or relevant to study findings.
Population characteristics	All subjects were between the ages of 18 and 25. Subjects were pre-screened for participation with a questionnaire to ensure volunteers had no medical implants, and were not pregnant or breastfeeding.
Recruitment	Participants were recruited from emails sent to collegiate club sports teams to target a study population of 'healthy' young adults. Healthy defined by lack of disorders affecting fluid balance or muscle content (sarcopenia, muscular dystrophy, kidney disease ,liver disease, or congestive heart failure.). Young athletes of both sexes were targeted as the study population to target a homogeneous population. A study population of young adults participating in athletics bias for a population with lower BMI, which may impact results as subjects may have different proportions of muscle mass and subcutaneous fat.
Ethics oversight	MIT Committee on the Use of Humans as Experimental Subjects (protocol 2002000099)

Note that full information on the approval of the study protocol must also be provided in the manuscript.

Field-specific reporting

Please select the one below that is the best fit for your research. If you are not sure, read the appropriate sections before making your selection.

☒ Life sciences ☐ Behavioural & social sciences ☐ Ecological, evolutionary & environmental sciences

For a reference copy of the document with all sections, see nature.com/documents/nr-reporting-summary-flat.pdf

Life sciences study design

All studies must disclose on these points even when the disclosure is negative.

Sample size	No statistical test was used to determine appropriate sample size prior to data collection. Sample size was determined based on SNR of data collected, which directly impacts the confidence in fitting methods used. For studies with replicate measurements of the same imaging phantom, a sample size of three 10 min acquisitions was used. For studies involving discrete samples (human subject and animal tissue) each experimental group consisted of 5 individual samples, or 5 individual subjects. All data met a minimum SNR requirement.
Data exclusions	No data was excluded from analysis in this manuscript. Data obtained with a signal-to-noise ratio of 20 or less would be excluded from analysis.
Replication	Standardized imaging phantoms were used to evaluate data reproducibility. Data was collected over the course of several weeks, in multiple locations (laboratory, athletic facility, hospital) as human subjects consented to study participation and as animal tissue became available for harvesting. Prior to data collection, ambient noise was measured and efforts taken to minimize noise (through grounding). Data reproducibility is higher when root mean square noise levels were below 0.3uV and signal to noise ratio is above 150. Data presented is an average of at least 3 trials with error bars present to capture variability.
Randomization	Data collection was performed randomly where possible. For studies involving imaging phantoms, care was taken to randomize the order of data collection by varying the phantom order and acquisition frequency to ensure replicates were not completed back-to-back. For studies involving animal tissue and human subjects, data was collected on the bases of subject recruitment and availability and animal tissue harvesting schedules.
Blinding	Blinding was not possible in this study. The study provides a characterization of device design and performance, for figures that demonstrate statistical significance between groups, the samples measured had very different physical characteristics and data could not be obtained blinded.

Reporting for specific materials, systems and methods

We require information from authors about some types of materials, experimental systems and methods used in many studies. Here, indicate whether each material, system or method listed is relevant to your study. If you are not sure if a list item applies to your research, read the appropriate section before selecting a response.

Materials & experimental systems

n/a	Involved in the study
☒	☐ Antibodies
☒	☐ Eukaryotic cell lines
☒	☐ Palaeontology and archaeology
☐	☒ Animals and other organisms
☒	☐ Clinical data
☒	☐ Dual use research of concern
☒	☐ Plants

Methods

n/a	Involved in the study
☒	☐ ChIP-seq
☒	☐ Flow cytometry
☒	☐ MRI-based neuroimaging

Animals and other research organisms

Policy information about studies involving animals; ARRIVE guidelines recommended for reporting animal research, and Sex and Gender in Research

Laboratory animals	Rat, Sprague Dawley, M, 5 months; Rat, WISTAR, F, 18 months
Wild animals	No wild animals were used in this study
Reporting on sex	No information on sex was collected. There is not expected to be a difference in findings based on sex and the findings of the study do not only apply to one sex.
Field-collected samples	No field collected samples were used in this study
Ethics oversight	Massachusetts Institute of Technology Committee on Animal Care, protocol 2208000409

Note that full information on the approval of the study protocol must also be provided in the manuscript.

Plants

Seed stocks	n/a
Novel plant genotypes	n/a
Authentication	n/a
